# Current manufactured cigarette smoking and roll-your-own cigarette smoking in Thailand: findings from the 2009 Global Adult Tobacco Survey

**DOI:** 10.1186/1471-2458-13-277

**Published:** 2013-03-27

**Authors:** Sarunya Benjakul, Lakkhana Termsirikulchai, Jason Hsia, Mondha Kengganpanich, Hataichanok Puckcharern, Chitrlada Touchchai, Areerat Lohtongmongkol, Linda Andes, Samira Asma

**Affiliations:** 1 Bureau of Tobacco Control, Department of Disease Control, Ministry of Public Health, Nonthaburi, Thailand; 2 Faculty of School of Public Health, Mahidol University, Bangkok, Thailand; 3 Office on Smoking and Health, Centers for Disease Control and Prevention, Atlanta, GA, USA; 4 National Statistical Office, Bangkok, Thailand

**Keywords:** Thailand, Manufactured cigarettes, Roll-your-own cigarettes (RYO), Prevalence of current smoking

## Abstract

**Background:**

Current smoking prevalence in Thailand decreased from 1991 to 2004 and since that time the prevalence has remained flat. It has been suggested that one of the reasons that the prevalence of current smoking in Thailand has stopped decreasing is due to the use of RYO cigarettes. The aim of this study was to examine characteristics of users of manufactured and RYO cigarettes and dual users in Thailand, in order to determine whether there are differences in the characteristics of users of the different products.

**Methods:**

The 2009 Global Adult Tobacco Survey (GATS Thailand) provides detailed information on current smoking patterns. GATS Thailand used a nationally and regionally representative probability sample of 20,566 adults (ages 15 years and above) who were chosen through stratified three-stage cluster sampling and then interviewed face-to-face.

**Results:**

The prevalence of current smoking among Thai adults was 45.6% for men and 3.1% for women. In all, 18.4% of men and 1.0% of women were current users of manufactured cigarettes only, while 15.8% of men and 1.7% of women were current users of RYO cigarettes only. 11.2% of men and 0.1% of women used both RYO and manufactured cigarettes. Users of manufactured cigarettes were younger and users of RYO were older. RYO smokers were more likely to live in rural areas. Smokers of manufactured cigarettes appeared to be more knowledgeable about the health risks of tobacco use. However, the difference was confounded with age and education; when demographic variables were controlled, the knowledge differences no longer remained. Smokers of manufactured cigarettes were more likely than dual users and those who used only RYO to report that they were planning on quitting in the next month. Users of RYO only appeared to be more addicted than the other two groups as measured by time to first cigarette.

**Conclusions:**

There appears to be a need for product targeted cessation and prevention efforts that are directed toward specific population subgroups in Thailand and include information on manufactured and RYO cigarettes.

## Background

In Thailand, more than 20 years of tobacco control regulations and laws [1,2], may have contributed, at least in part to decreasing the prevalence of smoking and exposure to secondhand smoke [3]. There has been a reduction in the prevalence of current smoking among men from 59.3% in 1991 to 41.7% in 2007 [3], while the prevalence among women has continued to remain low. However, there does not appear to have been any substantial decrease in prevalence since that time; current smoking among male adults in 2009 still remains relatively high at 40.5% [4].

Sangthong et al. [5] reported that the prevalence of current smoking in Thailand decreased from 1991 to 2004 and subsequently has remained flat. During this period, the rate of decline in the prevalence of manufactured cigarette smoking decreased later in the period. However, the prevalence of roll-your-own (RYO) cigarette smoking has remained flat since 1996. Young et al. [6] compared cigarette smokers in Thailand and in Malaysia in 2005, and found that many factors may play a role in tobacco use including taxation, and socio-cultural and economic factors, which may not act independently. “Economic motives are obviously extremely important, because RYO cigarettes are typically less expensive than FM (factory manufactured, added by the current authors) cigarettes and serve as a “discount” option for smokers” (Young et al. [6]). They also reported that any RYO use was associated with living in rural areas, older average age, lower level of education, being male, not working for pay, greater social acceptability of smoking, and attitudes toward tobacco regulation.

One of the purposes of this study was to provide an update of the information presented by Young et al. [6]. The stability of the findings is important given differences in the time period, sampling strategies and question wording. We were interested in determining whether similar patterns related to the demographic variables emerged after 4 years.

1. What is the prevalence of current tobacco use for males and females by type of cigarettes (manufactured vs RYO)? Do many people use both types of products (dual use)?

2. Are there differences in current use by age, by geographic location, by socio-economic status?

3. Are there differences in knowledge, addiction, and in desire to quit among different groups of users?

## Methods

From 2008 to 2010, 14 countries, most of them being either low or middle income with a heavy burden of tobacco use and highly populated, conducted the Global Adult Tobacco Survey (GATS) to monitor tobacco use in their countries and the changes driven by policies enacted in those nations [7]. Selected as one of the 14 countries, Thailand conducted GATS in 2009 [8,9]. To ensure useful comparisons across the 14 countries, the survey followed the standard GATS protocol.

The target population was noninstitutionalized residents, in Thailand, aged 15 years or above (defined as adults in the current study), and the sample was a nationally and regionally representative probability sample. A stratified three-stage cluster sampling design was used with the strata being five geographic regions: the Bangkok metropolitan area and the North, Northeast, Central, and South regions of Thailand which were further stratified into urban and rural areas. The sampling frame used was supplied by the National Statistical Office (NSO), derived from Thailand’s National Population and Housing Census 2000 [10]. Primary sampling units (PSUs) were blocks in urban areas and villages in rural areas and were randomly selected, in the first stage, using selection probability proportional to size sampling. In the second stage, 16 and 28 households were randomly chosen from the previously selected urban or rural PSUs, respectively, using simple systematic sampling. In the third stage, a face-to-face screening interview was used at each randomly selected household, in which a list of all eligible individuals in the household was drawn up and one person from the list was randomly selected to participate in the interview, using an algorithm of simple random sampling on the handheld device designed for data collection. If the respondent was not available, the interviewer would schedule another appointment to interview. Three attempts were made before the individual was considered a nonrespondent. If a residence was found to be empty, it was declared to be ineligible; if a selected respondent refused to participate, the individual was considered to be a nonrespondent.

All interviewees were informed that they could stop the survey and the response was confidential. Interviewers were selected by provincial statistical offices (PSO) and were only responsible for their own province, with supervisors from corresponding PSOs. Both interviewers and supervisors had experience on national health related surveys fieldwork. All of them had at least a Bachelor’s degree in education. There were 2 interviewers/supervisors - training sessions using the same trainers with technical support from CDC and WHO experts.

The interview was conducted in the selected household; while other members of the household could be present, they were instructed to remain silent during the interview. No proxy responding was allowed and no incentives were provided for participation. For the questionnaire interview (average duration was 19.1 minutes, SD = 7.6 minutes), the trained interviewers used personal digital assistants (PDAs) to collect data. The fieldwork started on February 1, 2009, and was completed on May 31 of the same year.

The questionnaire included information on demographics (age, gender, education, income); use of smoking tobacco and of smokeless tobacco; cessation attempts and interest in quitting; exposure to secondhand smoke; purchase of cigarettes during the last 30 days; exposure to media that provided information on smoking and health effects; questions about pictorial warning labels on manufactured cigarette packs, and knowledge, attitudes, and perceptions about tobacco use. Some questions were added by the Thailand GATS working group and reviewed by the Thailand GATS expert committee [9]. GATS protocols were approved by the Ethics Committee of the Faculty of Public Health, Mahidol University.

### Definition of variables

Used as an indicator of socioeconomic status, total personal income was grouped as low (less than 4,780 baht or less than 140.6 USD per month), middle (4,780 to 7,000 baht or 140.6 to 205.9 USD per month), and high (7,001 baht or higher or 206.0 USD or higher per month). Education was grouped into none or less than primary school, primary school, secondary school and university and above. RYO cigarettes, also known as hand-rolled cigarettes are referred to the cigarettes made by hand using shredded tobacco and papers. Current smoking was defined as responding yes to a question about smoking daily or less than daily. Whether smoking can cause seven well proved diseases, including stroke, heart stack, lung cancer, mouth cancer, larynx cancer, impotence, emphysema, was used as information to assess the extent of participants knowledge of health effects. A knowledge score, defined as the total number of the seven knowledge items answered correctly, was used as a summary measure of knowledge.

### Analysis

The data were reviewed for inconsistencies and out of range responses, edited, and weighted, using the complex survey analysis module of SPSS Version 18. Weights were computed as a product of three components: base weight, which was an inverse of the final probability of selection, adjustment for nonresponse at both the household and individual level, and post-stratification adjustment based on residence (urban or rural), age, and gender from the 2008 population projection for Thailand. A two sample test was used for pair comparison of prevalence among different group of users at statistically significant level of p < 0.05. Analyses of knowledge, addiction, and quitting, as well as multivariate analysis, were carried out for men only, because the levels of use of the tobacco products among females were too low for reliable analyses. Multivariate analyses, taking the complex sample design into account, used logistic regression models to see if the type of cigarettes smoked (manufactured cigarette only, RYO only and dual use) was associated with age, education, income, residence, and region. These variables were selected because of previous studies that have shown these variables to be related to tobacco use and the use of RYO. In the multivariate models, we determined the relationship of one variable while controlling for the effects of the others. The test for importance of a factor was carried out by a comparison of the full model with all factors and a reduced model where the factor of interest was excluded.

## Results

A total of 20,566 people aged 15 and over were surveyed in Bangkok and in the four regions of Thailand: North, Northeast, Central and South. The household-level response rate was 97.9%, the individual-level response rate was 96.2%, and the overall response rate, a product of household response rate and individual response rate, was 94.2%. Each of the four non-Bangkok regions had high response rates, with at least 95.1% overall, at least 98.4% at the household level, and at least 96.2% at the individual level. The response rate in the Bangkok metropolitan area was 94.6% at the household level, 90.0% at the individual level, and 85.1% overall.

### Prevalence of smoking of tobacco products

The prevalence of current smoking for all tobacco products (data not shown) was 45.6% among men and 3.1% among women. Although a variety of tobacco products were used including smokeless tobacco, pipes, cigars, and water pipes, 86.3% of all current tobacco users predominantly used two major products, manufactured cigarettes (54.9% of users) and RYO cigarettes (51.6% of users), and 20.2% of users smoked both (data not shown). Among smokers of any tobacco products, 40.4% of them smoked manufactured cigarettes only, 36.6% of them smoked RYO cigarettes only, and 23.4 of them were dual users (40.5%, 34.8%, and 24.7% respectively for men, 35.7%, 60.7%, and 3.6% respectively for women). As shown in Table 1, at a population level, 18.4% of men and 1.0% of women were current smokers of manufactured cigarettes only, while 15.8% of men and 1.7% of women were current smokers of RYO cigarettes only. For men, 11.2% used both manufactured and RYO. Among women, only 0.1% used both products. Among the dual users, 33.7% of men and 63.0% of women smoked more RYO cigarettes than did manufactured cigarettes (Table 1).

**Table 1:** Prevalence (in percent and 95%CI ) of current smoking by classification of product and demographic characteristics

**Male**								**Female**						
	**MC only**^**1**^		**RYO only**^**2**^		**Dual use**		RYO > MC*	**MC only**^**1**^		**RYO only**^**2**^		**Dual use**		RYO > MC*
Total	18.4	(17.3, 19.6)	15.8	(14.6, 17.2)	11.2	(9.9, 12.5)	33.7	1.0	(0.8, 1.2)	1.7	(1.4, 2.1)	0.1	(0.1, 0.2)	63.0
Age (in years)														
15–24	20.6	(17.0, 24.7)	3.2	(1.9, 5.5)	13.6	(10.6, 17.3)	32.9	0.9	(0.5, 1.6)	0.2	(0.0, 1.1)	0.0	(0.0, 0.3)	100.0
25–44	23.3	(21.4, 25.2)	14.8	(13.0, 16.8)	13.4	(11.6, 15.4)	32.3	1.1	(0.8, 1.5)	0.9	(0.6, 1.5)	0.2	(0.1, 0.4)	75.2
45–64	14.8	(13.3, 16.4)	21.5	(19.2, 23.9)	9.0	(7.4, 10.8)	38.2	1.2	(0.9, 1.6)	2.7	(2.0, 3.6)	0.2	(0.1, 0.4)	41.6
65+	6.7	(5.6, 8.0)	28.6	(25.7, 31.7)	4.5	(3.5, 5.9)	34.9	0.6	(0.3, 1.0)	3.9	(2.9, 5.2)	0.0	(0.0, 0.1)	0.0
Education														
None^3^	8.9	(7.8, 10.1)	31.8	(29.5, 34.2)	9.0	(7.5, 10.8)	38.7	0.9	(0.7, 1.2)	3.9	(3.1, 4.8)	0.1	(0.0, 0.2)	67.3
Primary	16.8	(14.8, 19.0)	19.0	(16.2, 22.2)	17.3	(14.6, 20.4)	39.7	1.0	(0.7, 1.6)	1.0	(0.5, 1.7)	0.3	(0.1, 0.7)	46.6
Secondary	25.8	(23.4, 28.3)	5.6	(4.3, 7.3)	11.6	(9.7, 13.8)	27.4	1.2	(0.8, 1.8)	0.1	(0.0, 0.4)	0.1	(0.0, 0.4)	81.2
University	22.7	(19.6, 26.1)	1.9	(1.2, 2.8)	3.9	(2.5, 6.0)	10.2	0.7	(0.3, 1.4)	0.0		0.0		
Income														
Low	11.7	(10.1, 13.6)	22.1	(20.0, 24.3)	10.3	(8.5, 12.4)	33.7	0.6	(0.4, 0.8)	2.4	(1.9, 3.0)	0.1	(0.0, 0.2)	63.1
Middle	21.9	(19.2, 24.8)	15.4	(13.3, 17.8)	15.8	(13.1, 18.8)	35.3	1.5	(1.1, 2.1)	1.4	(1.0, 2.1)	0.2	(0.1, 0.6)	71.5
High	24.1	(22.4, 25.9)	8.8	(7.5, 10.3)	9.4	(7.9, 11.1)	32.0	1.5	(1.1, 2.2)	0.5	(0.3, 1.0)	0.2	(0.1, 0.5)	54.7

^1 ^Smoked manufactured cigarettes only.

^2 ^Smoked roll-your-own cigarettes only.

^3 ^None or less than primary school.

* Percentage of dual users who smoked more roll-your-own cigarettes than manufactured cigarettes.

### Demographic variables

There were differences in product use by education and income levels (Table 1). Among men who used manufactured cigarettes only, the prevalence of use was highest in those aged 25–44 years (23.3%). Among men who used RYO cigarettes only, the highest prevalence was found in those aged 65 years or above (28.6%) for men, with the second highest prevalence group being those aged 45–64 (21.5%). Among women, the highest use of RYO cigarettes only was among those aged 45 or above. Dual use for men occurred most often in those under age 45. For both genders, adults with lower education levels or with lower incomes were more likely to smoke RYO cigarettes only or use both products than were those with university education (p < .001 for men and women) or high income (p < .001 for men and women). In the case of manufactured cigarettes, both men and women with middle or high income or secondary school or university level of education were more likely to smoke these products than those with low income (p < .001 for men and women) and those with primary school or less level of education (p < .001 for men and women). Across regions (Table 2), RYO cigarettes were more likely to be smoked in rural than in urban areas (p < .001), particularly in the South.

**Table 2:** Prevalence (in percent and 95% CI) of men’s current smoking by type of product, residence and region

**Urban**							**Rural**					
**Region**	**Manufactured cigarettes only**		**Roll-your-own cigarettes only**		**Dual use**		**Manufactured cigarettes only**		**Roll-your-own cigarettes only**		**Dual use**	
Bangkok	33.0	(30.3, 35.7)	2.8	(2.1, 3.8)	2.2	(1.6, 3.1)						
Central	29.7	(26.3, 33.3)	7.0	(5.3, 9.2)	4.3	(3.2, 5.8)	21.5	(18.6, 24.7)	15.4	(12.4, 19.0)	8.2	(5.8, 11.5)
North	24.1	(20.4, 28.3)	9.3	(6.9, 12.4)	2.7	(1.7, 4.4)	15.0	(11.8, 18.8)	20.1	(16.7, 24.0)	5.0	(3.5, 7.3)
Northeast	30.3	(26.6, 34.4)	8.8	(6.6, 11.6)	10.3	(7.8, 13.4)	10.1	(7.8, 13.0)	20.9	(17.7, 24.4)	16.0	(12.8, 19.9)
South	23.3	(19.8, 27.1)	12.6	(10.1, 15.6)	14.1	(11.5, 17.1)	9.0	(6.7, 12.0)	22.9	(19.4, 26.7)	28.0	(23.7, 32.7)
All	29.5	(27.9, 31.1)	6.8	(6.0, 7.7)	5.5	(4.8, 6.3)	13.6	(12.1, 15.3)	19.7	(18.0, 21.6)	13.6	(11.9, 15.5)

Table 3 shows the multivariate analysis of pairwise comparison of three categories of smokers: manufactured cigarette only, RYO cigarette only, and dual users, with demographic variables, age, education, income, residence, and region. In the comparison of smokers between RYO cigarette only and manufactured cigarette only, all five factors studied were statistically significant. Older age, lower education level, lower income, rural residence, and living in the South region were associated with being a RYO smoker. Similarly, in the comparison of smokers between RYO cigarette only and the dual users, older age, lower education, lower income, and living in the Central and North region, were associated with being a RYO smoker, while rural residence was not. Finally, in the comparison between the dual users and manufactured cigarette only, lower education, lower income, rural residence, living in the South region were associated with being a dual user, while age was not a significant factor.

**Table 3:** Multivariate analysis of type of smokers with demographic variables

**Variable**	**RYO only vs. Manufactured only**		**RYO only vs. Dual use**		**Dual use vs. Manufactured only**	
Age	p < 0.001*		p < 0.001*		p = 0.891*	
15–24	1.00		1.00		1.00	
25–44	4.85	(2.6, 8.9)	3.81	(1.9, 7.4)	1.03	(0.7, 1.6)
45–64	6.94	(3.6, 13.3)	6.03	(2.9, 12.6)	1.00	(0.6, 1.7)
65+	12.02	(6.1, 23.6)	12.68	(5.9, 27.2)	0.86	(0.5, 1.5)
Education	p < 0.001*		p < 0.001*		p < 0.001*	
None or primary school	22.21	(12.1, 40.9)	2.70	(1.4, 5.2)	7.18	(4.1, 12.6)
Primary	12.50	(6.8, 23.1)	1.88	(1.0, 3.5)	6.47	(3.7, 11.2)
Secondary	2.90	(1.6, 5.4)	1.05	(0.6, 2.0)	2.80	(1.7, 4.7)
University	1.00		1.00		1.00	
Income	p < 0.001*		p < 0.010*		p = 0.074*	
Low	3.96	(2.8, 5.5)	1.81	(1.3, 2.6)	1.49	(1.0, 2.3)
Middle	1.73	(1.3, 2.3)	1.00	(0.7, 1.3)	1.46	(1.0,2.1)
High	1.00		1.00		1.00	
Residence	p < 0.001*		p = 0.438*		p < 0.001*	
Rural	3.94	(3.0, 5.2)	1.13	(0.8, 1.5)	3.52	(2.6, 4.7)
Urban	1.00		1.00		1.00	
Region	p < 0.001*		p < 0.001*		p < 0.001*	
Bangkok	0.08	(0.0, 0.1)	1.40	(0.8, 2.4)	0.07	(0.0, 0.1)
Central	0.17	(0.1, 0.3)	1.49	(1.0, 2.3)	0.12	(0.1, 0.2)
North	0.20	(0.1, 0.3)	2.40	(1.6, 3.7)	0.09	(0.1, 0.2)
Northeast	0.41	(0.2, 0.7)	1.02	(0.7, 1.5)	0.43	(0.3, 0.7)
South	1.00		1.00		1.00	

*p-value of the test for importance of the factor.

### Knowledge

Table 4 presents the results of the univariate analyses. The percent of males who answered all 7 knowledge questions correctly was calculated, with 57.7% who smoked manufactured cigarettes only and 49.0% who smoked RYO (p = 0.003). There was no difference between the dual users and those who used manufactured cigarettes only. After controlling for age, education, income, residence, and region, we did not find statistically significant differences between RYO only and manufactured cigarette only smokers (p = .194). A similar pattern of results was found when the knowledge scores were examined. In the univariate analysis, those who smoked manufactured cigarettes only had the higher average knowledge score than those who used RYO only (6.1 vs. 5.5, p < .001). After controlling for age, education, income, residence, and region, the p-value became 0.11.

**Table 4:** Percentage (95% CI) of men’s daily smokers who have correct knowledge of smoking causing diseases

	**Manufactured**		**Roll-your-own**		**Dual users**	
**Diseases**	**Cigarette smokers only**		**Cigarette smokers only**			
Stroke	83.3	(80.4, 85.9)	73.3	(69.2, 77.1)	82.7	(77.5, 87.0)
Heart Attack	76.6	(73.4, 79.6)	69.3	(65.3, 73.1)	74.6	(68.7, 79.8)
Lung Cancer	97.1	(95.5, 98.1)	94.5	(92.6, 96.0)	97.3	(95.7, 98.4)
Mouth Cancer	93.6	(91.8, 95.0)	85.9	(82.7, 88.6)	92.8	(90.3, 94.6)
Larynx Cancer	94.8	(93.0, 96.1)	85.7	(82.4, 88.5)	92.2	(88.3, 94.9)
Impotence	70.4	(67.1, 73.5)	59.4	(55.2, 63.5)	72.9	(66.9, 78.2)
Emphysema	92.8	(90.0, 94.9)	84.7	(81.1, 87.7)	87.9	(82.7, 91.6)
Answer all above questions correctly	57.7	(54.0, 61.4)	49.0	(44.5, 53.4)	59.0	(53.0, 64.7)
Knowledge score*	6.1	(5.9, 6.2)	5.5	(5.3, 5.7)	6.0	(5.8, 6.2)

*The number of knowledge questions correctly answered.

### Addiction and quitting

Table 5 presents the results of analyses of addition. Time to the first smoking of a cigarette after waking can be used as an indicator of nicotine dependence. Overall, a higher percentage of smokers of RYO cigarettes were significantly more likely to report smoking their first cigarette within 30 minutes after waking up than smokers of manufactured cigarettes (64.3% vs. 57.6%, p = .018).

**Table 5:** Percentage (95% CI) of men daily smokers who smoke within 30 minutes of awakening by demographic characteristics

	**Manufacturedcigarette smokers only**		**Roll-your-owncigarette smokers only**		**Dual users**	
Total	57.6	(53.9, 61.2)	64.3	(60.0, 68.4)*	60.4	(54.3, 66.1)
Age (in years)						
15–24	44.7	(34.1, 55.7)	61.4	(31.8, 84.4)	51.6	(36.4, 66.4)
25–44	62.4	(57.4, 67.1)	66.1	(59.0, 72.6)	60.4	(52.7, 67.6)
45–64	58.8	(53.0, 64.5)	67.0	(61.6, 72.0)*	70.0	(62.6, 76.4)**
65+	51.3	(41.5, 60.9)	58.7	(52.8, 64.5)	64.8	(50.4, 76.9)
Education						
none or less than primary school	54.7	(47.3, 62.0)	64.7	(60.0, 69.1)*	66.3	(57.3, 74.2)**
Primary	60.8	(53.6, 67.5)	65.3	(55.7, 73.8)	57.4	(47.4, 66.9)
Secondary	56.5	(50.6, 62.3)	60.1	(46.5, 72.3)	59.8	(49.6, 69.2)
University	58.8	(49.4, 67.7)	64.1	(42.1, 81.5)	52.8	(33.1, 71.7)
Income						
Low	50.7	(41.8, 59.6)	65.2	(59.5, 70.4)*	55.4	(46.5, 64.0)
Middle	57.3	(50.2, 64.1)	63.4	(55.8, 70.4)	61.6	(51.3, 71.0)
High	61.1	(56.7, 65.3)	62.7	(54.4, 70.4)	65.0	(55.5, 73.4)
Region						
Bangkok	64.8	(59.5, 69.8)	73.7	(59.2, 84.4)	61.7	(45.1, 76.0)
Central	59.8	(53.2, 66.0)	67.3	(60.7, 73.2)	66.4	(56.9, 74.7)
North	53.6	(44.3, 62.5)	65.0	(56.5, 72.6)*	60.2	(42.9, 75.2)
Northeast	50.8	(41.4, 60.0)	61.3	(52.6, 69.4)	60.2	(49.1, 70.4)
South	58.9	(49.8, 67.5)	66.3	(59.3, 72.6)	57.4	(49.1, 65.4)

* p-value < 0.05 of the test for difference between roll-your-own cigarette only smokers and manufactured cigarette smokers.

** p-value < 0.05 of the test for difference between dual users and manufactured cigarette smokers.

As shown in Table 6, among men, smokers of manufactured cigarettes were more likely to report plans to quit within the next month than were men who RYO cigarettes (7.8% vs. 4.4% and 4.2% for dual users p < 0.001), and overall men who smoked RYO were more likely to report not being interested in quitting than were those who smoked manufactured cigarettes. A multivariate analysis (not shown) adjusting for age, education, income, residence and region showed the same pattern (p = .005).

**Table 6:** Percentage (95% CI) of men daily smokers who intent to quit smoking by demographic characteristics

	**Manufactured cigarette smokers only**		**Roll-your-own cigarette smokers only**		**Dual users**	
Plan to quit within next month	7.8	(6.2, 9.8)	4.4	(3.2, 6.0)*	4.2	(2.8, 6.2)**
Think about quitting within next 12 months	17.2	(14.5, 20.3)	12.6	(10.3, 15.3)*	17.4	(13.3, 22.5)
Will quit someday but not in next 12 months	35.9	(32.5, 39.5)	36.6	(32.7, 40.7)	43.3	(37.7, 49.1)**
Not interested in quitting	34.3	(30.7, 38.1)	42.8	(38.6, 47.0)*	32.8	(27.8, 38.3)
Don’t know	4.7	(3.4, 6.5)	3.7	(2.4, 5.6)	2.2	(1.3, 3.9)**
Total	100.0		100.0		100.0	

* p-value < 0.05 of the test for difference between roll-your-own cigarette only smokers and manufactured cigarette smokers.

** p-value < 0.05 of the test for difference between dual users and manufactured cigarette smokers.

## Discussion

In 2009, we found that the prevalence of current smoking among Thai adults was 45.6% for men and 3.1% for women. In all, 18.4% of men and 1.0% of women were current users of manufactured cigarettes only, while 15.8% of men and 1.7% of women were current users of RYO cigarettes only and 11.2% of men and 0.1% of women used both RYO and manufactured cigarettes. Users of manufactured cigarettes were younger and users of RYO were older. RYO smokes were also more likely to live in rural areas. When the demographic variables were controlled, there were no differences between the three groups of smoking either in the percentage who answered all seven questions correctly or in total knowledge score. Respondents who smoked only manufactured cigarettes were more likely than dual users and those who used only RYO to report that they were planning on quitting in the next month. Users of RYO only appear to be more addicted than the other two groups as measured by time to first cigarette. These analyses provide recent estimates than have been reported and present information on knowledge and addiction that has not been reported in earlier work.

While comparisons with other surveys such as that National Cigarette Smoking and Alcohol Drinking Survey and the ITC survey can be made, caution should be exercised in that sampling and question wording differed. However, the findings from this study closely mirror those from Young et al. [6] who found that any RYO use was associated with living in rural areas, older average age, lower level of education, male gender, not being in paid work, slightly lower consumption of cigarettes, higher social acceptability of smoking, and positive attitudes toward tobacco regulation.

The proportion of RYO cigarette smokers is higher in Thailand than in its neighbors, such Vietnam (1.1%) and China (2.3%) [11,12]. As Young et al. [6] noted there are many factors that may play a role in differences in prevalence in different countries including cultural, legislative and economic factors.

One important factor in stimulating the decrease in the prevalence of current smoking in Thailand can be found in the increase in the taxation of manufactured cigarettes [13,14]; the excise tax for manufactured cigarettes has increased from 55% in 1992 to 85% in 2009 [13]. However, the taxation rate for blended shredded tobacco, which is used in RYO cigarettes, has remained as low as 500 Thai Baht (approximately 16.5 U.S. dollars, per kilogram since October 13, 1999), leading to the possibility that some smokers of manufactured cigarettes may have started smoking more RYO cigarettes and reduced or eliminated their smoking of manufactured cigarettes.

Since there were differences noted in rural areas and for those with lower education and income, it may be that more focused education programs should be developed focusing on RYO smoking. Enhancement of health education programs with information on the harmful effects of both types of cigarettes may provide much needed information for smokers.

There are some limitations that should be noted about this survey. Questions on reasons for use of RYO were not included and, therefore, the explanations about economic and cultural facts are speculative. Given that smoking among women does not appear to be acceptable, the estimate of prevalence for women may be subject to some misreporting. Some of the strengths include the rigorous procedures for sampling and interviewing used in GATS Thailand and the high response rate.

Thailand has implemented comprehensive tobacco control interventions, including educational campaigns in the media, legislation to ban smoking in public places, increases in the cigarette excise tax, bans on advertising, and the use of a pictorial warning labels on manufactured cigarettes. Taken together, these legislative measures may have changed social norms and perceptions about tobacco use in Thailand. Meanwhile, lack of comparable policies in the control of RYO cigarettes may be playing a role in the overall prevalence of smoking in Thailand, diluting the success of existing programs.

## Conclusions

There are demographic differences among the three groups of male tobacco users. RYO smokers are older and more likely to live in rural areas. There appears to be a need for product targeted cessation and prevention efforts that are directed toward specific population subgroups in Thailand and include information on manufactured and RYO cigarettes. In addition, adoption of tobacco control measures for RYO products similar to those for manufactured cigarettes could be considered.

## Competing interest

All authors declare to have no competing interest.

## Authors’ contributions

SB and LT supervised the GATS Thailand and critically commented the manuscript. JH provided technical support for the GATS Thailand, analyzed the data and drafted the manuscript. MK, CT and AL contributed to the GATS Thailand operation. HP contributed to the sample design. LA contributed to the data analysis. SA critically reviewed the manuscript. All authors read and approved the final version of the manuscript.

## Pre-publication history

The pre-publication history for this paper can be accessed here:

http://www.biomedcentral.com/1471-2458/13/277/prepub

## References

[B1] Tobacco products control Act, B.E. 2535 1992 http://www.searo.who.int/fctc/reporting/Thailand_annex3_tobacco_act1992.pdf

[B2] Non-Smokers’ health protection Act 1992 http://thailaws.com/law/t_laws/tlaw0181.pdf23538783

[B3] Termsirikulchai L Benjakul S Kengganpanich M Theskayan N Nakju S Thailand tobacco control country profile 2008 Tobacco Control Research and Knowledge Management Center: Mahidol University

[B4] National Statistical Office, Thailand 2009 National health, welfare, and food consumption survey report 2009 Bangkok, Thailand: National Statistical Office

[B5] Sangthong R Wichaidit W Ketchoo C Current situation and future challenges of tobacco control policy in Thailand Tob Control 2011 21 49 54 21791510 10.1136/tc.2011.043331

[B6] Young D Yong HH Borland R Ross H Sirirassanmee B Kin F Prevalence and correlates of roll-your-own smoking in Thailand and Malaysia: findings of the ITC-south east Asia survey Nicotine Tob Res 2008 10 907 915 10.1080/14622200802027172 18569766

[B7] Kalsbeek WD Bowling JM Hsia J Mirza S Palipudi KM Asma S The Global Adult Tobacco Survey (GATS): sample design and related methods Proceedings of the section on survey methods, joint statistical meetings 2010 http://www.amstat.org/sections/srms/proceedings/y2010/Files/307559_58832.pdf

[B8] Hsia J Benjakul S Termsirikulchai L Puckcharern H Kengganpanich M For GTSS collaborative Group Methodology for Global Adult Tobacco Survey (GATS) in Thailand [abstract] Proceedings of Asian pacific conference on tobacco or health October 7, 2010 Sydney, Australia

[B9] World Health Organization, Regional Office for South‐East Asia Global adult tobacco survey (GATS) Thailand country report 2009 Bangkok, Thailand: Ministry of Health

[B10] National Statistical Office, Thailand National population and housing census 2000 http://web.nso.go.th/pop2000/pop_e2000.htm

[B11] Ministry of Health, Vietnam Global adult tobacco survey (GATS) Vietnam (2010) country report 2010 Hanoi, Vietnam: Ministry of Health

[B12] Yang GH Li Q Wang CX Hsia J Yang Y Xiao L Findings from 2010 Global Adult Tobacco Survey: implementation of MPOWER policy in China Biomedical and Environmental Science 2010 23 422 429 10.1016/S0895-3988(11)60002-0 21315239

[B13] Visaruthvong C Thailand tobacco tax report card 2011 http://www.smoke-free.ca/trade-and-tobacco/Thailand/Thailand%20Tax%20Report%20Card%202010.pdf

[B14] Levy DT Benjakul S Ross H Ritthiphakdee B The role of tobacco control policies in reducing smoking and deaths in a middle income nation: results from the Thailand SimSmoke simulation model Tob Control 2008 17 53 59 10.1136/tc.2007.022319 18218810

